# Meta-Analysis Reveals a Lack of Association between UGT2B17 Deletion Polymorphism and Tumor Susceptibility

**DOI:** 10.1371/journal.pone.0096812

**Published:** 2014-05-06

**Authors:** Xiaheng Deng, Yidong Cheng, Xiao Yang, Shuang Li, Ruizhe Zhao, Kang Liu, Jinliang Liu, Qiang Cao, Chao Qin, Pengfei Shao, Xiaoxin Meng, Jie Li, Qiang Lu, Changjun Yin

**Affiliations:** Department of Urology, the First Affiliated Hospital of Nanjing Medical University, Nanjing, China; Nanjing Medical University, China

## Abstract

**Purpose:**

UGT2B17 is a vital member of the UGT2 family and functions as a detoxification enzyme which catalyzes the glucuronidation of lipophilic compounds. Accumulating evidences implicates that it may contribute to the susceptibility of tumor risk. Identification of a *UGT2B17* deletion polymorphism has attracted studies to evaluate the association between the *UGT2B17* deletion polymorphism and tumor risk in diverse populations. However, the available results are conflicting.

**Methods:**

A meta-analysis based on 14 studies from 10 publications including 5,732 cases and 5,112 controls was performed. Published literature from PubMed, EMBASE and Web of Science was pooled and the crude odds ratios (ORs) with 95% confidence intervals (CIs) were calculated to estimate the strength of the associations.

**Results:**

Conclusively, our results indicate that individuals with a *UGT2B17* deletion polymorphism were associated with tumor risks (OR = 1.29, 95% CI = 1.03–1.63, *P*<0.001) in a recessive model. However, after excluding two studies for their heterogeneity, the result then demonstrated that the *UGT2B17* deletion polymorphism was not associated with tumor risks (OR = 1.118, 95%CI = 0.938–1.332, *P*>0.1). A subgroup analysis based on tumor type, sex or race did not show significant results.

**Conclusion:**

These results suggest that the *UGT2B17* deletion polymorphism is not associated with tumor risks.

## Introduction

Tumors are a multifactorial disease and have become a major global public health burden in recent years. However, the precise mechanism of carcinogenesis is complicated and still remains to be identified. Studies have demonstrated that tumors are a result of the intricate interactions between interior and environmental factors [1], such as race, age, family history, obesity, red and processed meat consumption, smoking and steroid hormone levels [2]. It has been demonstrated that enzymes may play an important role in the sensitivity to tumor risk through their functions of detoxifying carcinogenic substances and DNA synthesis [3], [4].

UDP-glucuronosyltransferases (UGTs) are a family of phase II detoxification enzymes which catalyze the glucuronidation of lipophilic compounds like drugs, dietary substances, tobacco toxins and environmental xenobiotics [5]–[7]. Additionally, UGTs function to glucuronidate endogenous and exogenous estrogens and androgens and then transfer them to less active compounds which might reduce the risk of sex-related tumors, such as prostate and breast tumor.

UGT2B17 is a vital member of the UGT2 family and it is ubiquitously expressed in hepatic tissues as well as kidney, skin, lung, breast, uterine, and prostate. Studies have showed that UGT2B17 may contribute to the progress of chronic lymphatic leukemia and has an impact on antineoplastic drug metabolism in tumor cells [8]. The human *UGT2B17* genes are clustered on chromosome 4q13 and have a high level of cDNA and amino acid sequence homology with other UGT2B enzymes [9], [10]. Previous studies have demonstrated a 150-kb deletion polymorphism that spans the whole *UGT2B17* gene [11], [12]. This polymorphism was found to significantly reduce the glucuronidation rates of several endogenous compounds in human liver microsomes [13]. Recently, genetic data has shown that the deletion polymorphism in *UGT2B17* is involved in the etiology of various tumors. An individuals' susceptibility to breast, lung, or prostate tumors might be associated with the *UGT2B17* null genotype, which does not express protein [14].

A number of studies have been done to elucidate the possible association between the *UGT2B17* null genotype and risks for different types of tumor in diverse populations, such as prostate tumors [15]–[20], lung tumors [10], [21], breast tumors [22], and colorectal tumors [23]. However, the results are inconclusive. Due to the imperative role of the *UGT2B17* deletion polymorphism in the carcinogenic process, we executed a meta-analysis on all accessible case-control studies to estimate the overall tumor risk associated with the polymorphism and to measure the potential between-study heterogeneity.

## Materials and Methods

### Identification of Relevant Studies

A comprehensive search of PubMed, EMBASE and Web of Science was conducted for relevant studies on the association between the *UGT2B17* null genotype and tumor susceptibility, covering all the papers published through October 15^th^, 2013. Publications were identified using the following search terms: “Uridine diphosphoglucuronosyl transferases 2B17”, “*UGT2B17* polymorphism”, “*UGT2B17* deletion”, “tumor” or “carcinoma”. Additional literature was collected from a hand search from the references of original studies identified or review articles. No language restrictions were imposed. Only the latest or more comprehensive sample size was included if the studies had overlapping subjects. Studies chosen had to meet the following inclusion criteria: (1) a case-control design and (2) contain available genotype frequency. The major exclusion criterion was duplicates of previous publications.

### Data extraction

Two reviewers independently extracted the data and reached a consensus on all items. The following information was obtained from each publication: author's first name, publication year, gender, tumor type, country of origin, ethnicity, number of cases and controls, source of control groups (population-based or hospital-based) and genotyping methods. The ethnic descents were classified as European, African, Asian and mixed. In four of the studies the data was presented as “Ins/Ins+ Ins/Del”, without the data for all three genotypes [15], [17], [19], [22]. In addition, the OR was calculated for the recessive model with statistical analysis.

### Statistical analysis

Hardy-Weinberg equilibrium (HWE) was evaluated using the goodness-of-fit chi-square test and a *p<0.05* was regarded to have a significantly selective bias [24]. Odds ratios (ORs) with 95% confidence intervals (CIs) were used to evaluate the strength of the association between the *UGT2B17* null genotype and tumor risk. A significantly increased or reduced tumor risk was defined as having a 95% CI without one for OR. The fixed-effects and random-effects models are based on the Mantel-Haenszel method, and the DerSimonian and Laird methods were respectively utilized to pool the data [25]. However, the random-effects model is more appropriate for one of the studies [19] that contains a much larger sample size than others studies and there was significant heterogeneity (*P*<0.001). First, the risks of the Del/Del and Ins/Del genotypes on tumors versus the wild-type Ins/Ins homozygote and (Ins/Del+ Del/Del) versus Ins/Ins from the data acquired was estimated. Next, the risks of the Del/Del versus (Ins/Ins+ Ins/Del) from all the pooled studies was estimated, assuming recessive effects of the *UGT2B17* Del polymorphism. Stratified analyses were performed in accordance with sex, tumor type and ethnicity. Heterogeneity between the studies was calculated with the χ^2^-based *Q* test and *P<0.05* was considered significant [26]. The presence of publication bias was examined using Begg's funnel plot and the Egger's linear regression test and *P<0.05* was considered significant [27]. All analyses were performed with Stata software (version 11.0; StataCorp LP, College Station, TX) with two-sided *P* values.

## Results

### Characteristics of accessible studies

**Figure 1:**
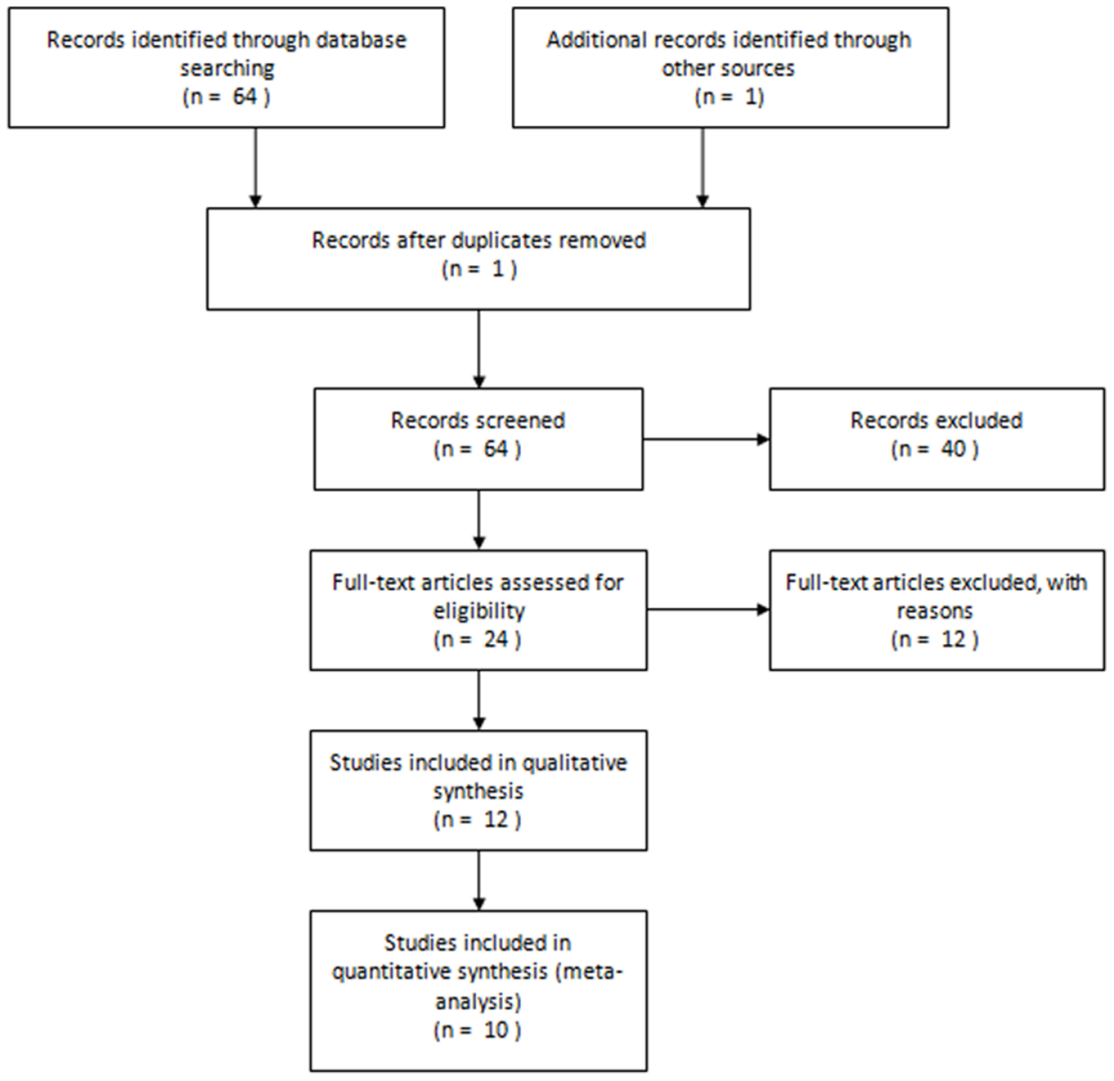
Flowchart of literature search and selection. A total of 14 studies from 10 published articles were identified in this meta-analysis.

The analysis included 14 studies from 10 publications on *UGT2B17* deletion polymorphism and tumor risk, including 5,732 cases and 5,112 controls, (Table 1). The flowchart of selection is shown in Figure 1. All studies were case-control studies with the following tumor type distribution: seven prostate tumor, four lung tumor, two colorectal tumor and one breast tumor. Ten of the studies were conducted in men and the reaming was conducted in on women. Eleven studies investigated the risks in populations of European descent and the other three studies investigated populations of African, Asian and mixed descents. Two genotyping methods were used: PCR-RFLP and Taqman. The controls used in eight of the studies were the healthy population, and patients hospitalized for reasons other than tumor in six of the studies. DNA was extracted from whole blood was used in thirteen of the studies and one study used DNA extracted from both blood and paraffin-embedded tissues. Tumors were confirmed histologically or pathologically in all studies. The distribution of the genotypes in the controls in the studies was in agreement with the HWE except for in five of the studies did not provide data for all three genotypes [15], [17], [19], [22].

**Table 1:** Characteristics of studies included in the meta-analysis.

ID	First author	Year	Sex	Tumor Type	Country	Ethnic Group	Material	Genotyping method	SOC	Case	Control	HWE
1	Park	2006	Male	Prostate tumor	USA	European	Blood	PCR-RFLP	Hospital	293	367	NA
2	Park	2006	Male	Prostate tumor	USA	African	Blood	PCR-RFLP	Hospital	112	115	NA
3	Gallagher	2007	Male	Prostate tumor	USA	European	Blood	Taqman	Hospital	411	397	Y
4	Park	2007	Male	Prostate tumor	USA	Mixed	Blood	PCR-RFLP	Hospital	246	273	NA
5	Carla	2007	Female	Lung tumor	USA	European	Blood	Taqman	Population	175	324	Y
6	Carla	2007	Male	Lung tumor	USA	European	Blood	Taqman	Population	223	373	Y
7	Karypidis	2008	Male	Prostate tumor	Sweden	European	Blood	Taqman	Hospital	174	161	Y
8	Olsson	2008	Male	Prostate tumor	Sweden	European	Blood	Taqman	Population	2682	1672	NA
9	Setlur	2010	Male	Prostate tumor	Austria	European	Blood	Taqman	Hospital	121	205	Y
10	Ebraham	2012	Female	Breast tumor	Iran	Asian	Mixed*	PCR-RFLP	Population	236	203	NA
11	Andrea	2013	Female	Colorectal tumor	USA	European	Blood	Taqman	Population	271	246	Y
12	Andrea	2013	Male	Colorectal tumor	USA	European	Blood	Taqman	Population	335	327	Y
13	Michaela	2013	Female	Lung tumor	Austria	European	Blood	Taqman	Population	131	142	Y
14	Michaela	2013	Female	Lung tumor	Austria	European	Blood	Taqman	Population	322	307	Y

PCR-RFLP: polymerase chain reaction –restriction fragment length polymorphism.

*Mixed: Materials for genotyping was extracted from blood and paraffin-embedded tissue specimens.

### Quantitative synthesis

**Figure 2:**
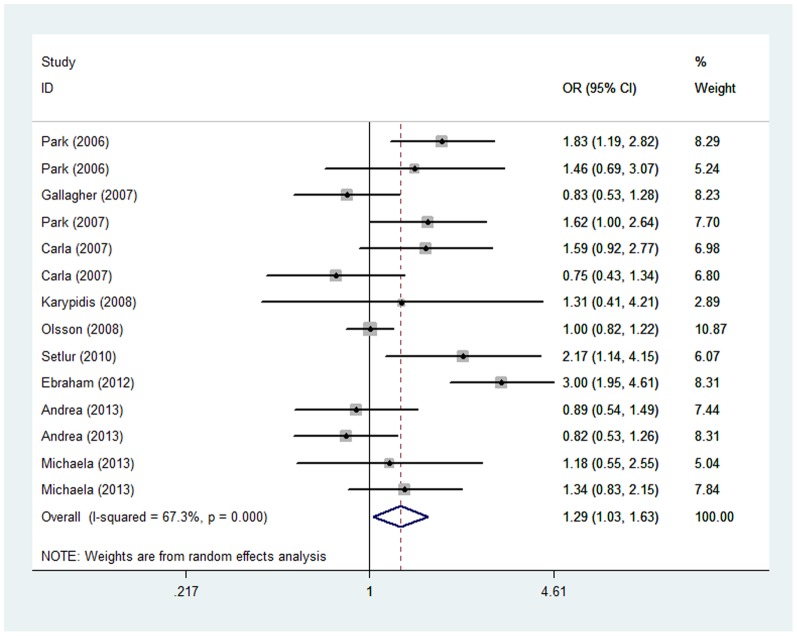
Forest plot of tumor risk for Del/Del versus Ins/Ins+Ins/Del. The squares and horizontal lines correspond to the study specific OR and 95% CI. The area of the squares reflects the study specific weight (inverse of the variance). The diamond represents the summary OR and 95% CI. OR, odds ratio; CI, confidence interval; I^2^, measure to quantify the degree of heterogeneity in the meta-analysis.

When the eligible studies had data for all three genotypes, they were pooled for the meta-analysis. The results showed there was no association between the variant genotypes and tumor risk in all three models (Ins/Ins versus Del/Del, Ins/Del versus Del/Del and the dominant model) (Table 2). In addition, the analysis failed to find any significant results on tumor risk according to specific tumor type, gender, or different control sources. Next, the effects of the recessive model was evaluated and the data demonstrated that individuals with the Del/Del genotype were associated with a statistically significant declined tumor risk (OR = 1.29, 95% CI = 1.03–1.63, *P*<0.001 for heterogeneity test). In a subgroup analysis on the source of control material, there was a significantly decreased risk in the hospital-based control group (OR = 1.46, 95% CI = 1.06–2.01, *P*>0.1 for heterogeneity test) using the recessive model. Nevertheless, the exclusion of two studies for their heterogeneity [15], [22] did not reveal an association between the *UGT2B17* null genotype and tumor risk (OR = 1.12, 95% CI = 0.94–1.33, *P*>0.1 for heterogeneity test) (Figure 2) (Table 3).

**Table 2:** Summary OR of the UGT2B17 null genotype and tumor risk for all the pooled studies.

	Comparisons	Cases/ controls	Ins/Ins versus Del/Del, OR(95%CI)^a^	*P* *	Ins/Del versus Del/Del, OR(95%CI)	*P* *	Dominant model (Ins/Del+ Del/Del versus Ins/Ins)^a^	*P* *		Comparisons	Cases/ control	Recessive model (Del/Del versus Ins/Ins+Ins/Del)^a^	*P* *
**Total**	9	2163/2482	0.823 (0.534–1.268)	<0.001	0.925 (0.763–1.122)	0.541	1.154 (0.823– 1.618)	<0.001	**Total**	14	5733/5112	**1.294** **(1.029–1.626)**	<0.001
**Sex**									**Sex**				
Male	6	1586/1770	0.770 (0.402–1.475)	<0.001	0.971 (0.773–1.220)	0.691	1.304 (0.784– 2.167)	<0.001	Male	10	4920/4197	1.189 (0.954–1.481)	0.024
Female	3	577/712	0.882 (0.617–1.261)	0.555	0.824 (0.575–1.180)	0.193	0.961 (0.768– 1.203)	0.875	Female	4	813/915	1.534 (0.854–2.752)	0.003
**Tumortype**									**Tumortype**				
Lung tumor	4	851/1146	0.917 (0.656–1.283)	0.291	0.761 (0.564–1.027)	0.382	0.877 (0.733– 1.050)	0.758	Lung tumor	4	851/1146	1.191 (0.864–1.640)	0.290
Prostate tumor	3	706/763	0.420 (0.083–2.126)	<0.001	1.073 (0.746–1.544)	0.645	2.437 (0.821– 7.233)	<0.001	Prostate tumor	7	4040/3190	1.329 (0.999–1.767)	0.025
Colorectal tumor	2	606/573	1.352 (0.919–1.990)	0.282	1.053 (0.744–1.491)	0.673	0.793 (0.479– 1.315)	0.033	Colorectal tumor	2	606/573	0.851 (0.612–1.183)	0.800
**Source of** **control**									Breast tumor	1	236/203	2.996 (1.946–4.613)	
Hospital	3	706/763	0.420 (0.083–2.126)	<0.001	1.073 (0.746–1.544)	0.645	2.437 (0.821–7.233)	<0.001	**Source of** **control**				
Population	6	1457/1719	1.063 (0.798–1.417)	0.175	0.875 (0.697–1.098)	0.392	0.842 (0.715–0.990)	0.274	Hospital	6	1358/1518	**1.456** **(1.056–2.007)**	0.106
									Population	8	4345/3594	1.198 (1.029–1.626)	<0.001
									**Ethnicity**				
									European	11	5138/4521	1.134 (0.929–1.384)	0.047
									African	1	113/115	1.458 (0.692–3.073)	
									Asian	1	236/203	2.996 (1.946–4.613)	
									Mixed	1	246/273	1.620 (0.996–2.636)	

Bold-faced values indicate significant difference.

*P value of Q test for heterogeneity.

a Random-effects model was used when *P* value for heterogeneity test <0.1; otherwise, fixed-effects model was used.

**Table 3:** Summary OR of the UGT2B17 null genotype and tumor risk after the elimination of the two studies by Ebraham *et al*. and Park *et al*.

	Comparisons	Cases/control	Recessive model (Del/Del versus Ins/Ins+Ins/Del)^a^	P*
**Total**	12	5203/4542	1.118(0.938–1.332)	0.136
**Sex**				
Male	9	4626/3830	1.112(0.901–1.372)	0.092
Female	3	577/712	1.172(0.817–1.682)	0.324
**Tumortype**				
Lung tumor	4	851/1146	1.191(0.864–1.640)	0.290
Prostate tumor	6	3746/2823	1.229(0.922–1.638)	0.082
Colorectal tumor	2	606/573	0.851(0.612–1.183)	0.800
**Source of control**				
Hospital	5	1064/1151	1.367(0.933–2.002)	0.119
Population	7	4139/3391	1.019(0.875–1.186)	0.399
**Ethnicity**				
European	10	4845/4154	1.057(0.883–1.265)	0.047
African	1	113/115	1.458(0.692–3.073)	
Mixed	1	246/273	1.620(0.996–2.636)	

Bold-faced values indicate significant difference.

*P value of Q test for heterogeneity.

a Random-effects model was used when *P* value for heterogeneity test <0.1; otherwise, fixed-effects model was used.

### Test of heterogeneity

**Figure 3:**
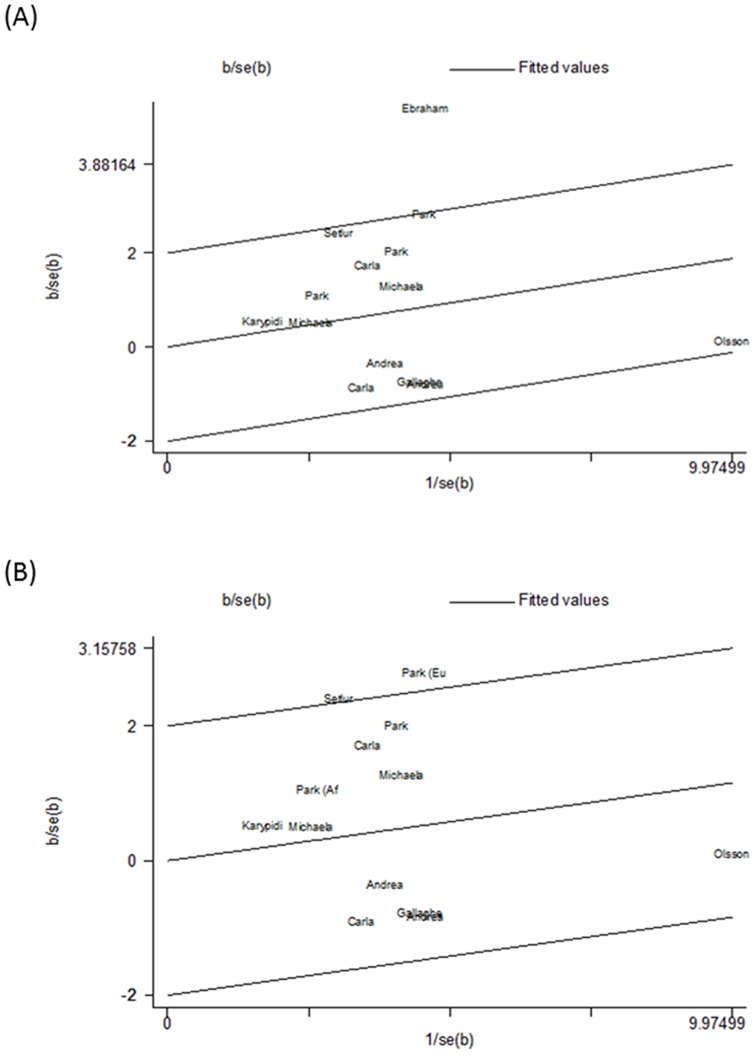
Galbraith radial plot for Del/Del versus Ins/Ins+Ins/Del. (A). The figure shows the contribution of individual studies to the heterogeneity and a study by Ebraham et al was found to be the cause of the heterogeneity. (B). After the exclusion of the study by Ebraham et al, the result of the contribution of individual studies to the heterogeneity.

Heterogeneity between studies was observed in the overall comparisons as well as in the subgroup analyses. There was significant heterogeneity in recessive genetic model (*P*<0.001). Meta-regression was utilized to evaluate the source of heterogeneity in the recessive model for gender (male or female) (*P* = 0.34), tumor type (lung tumor, prostate tumor, colorectal tumor or breast tumor) (*P* = 0.66), material (blood or mixed) (*P* = 0.02), control source (population-based or hospital based) (*P* = 0.43) and ethnicity (European, Asian, African and mixed) (*P* = 0.06). The results show that the material used for DNA extraction and the ethnicity did contribute to substantial altered heterogeneity. Next, a Galbraith radial plot was performed to delineate which study was the cause of the heterogeneity (Figure 3). After the elimination of one study [22], the results interestingly exhibited that the source of the control material prominently contributed to the heterogeneity of the present analysis (*P* = 0.01). Again, the Galbraith radial plot was utilized to identify another heterogeneous study, the Park *et al.* study, based on a European population, contributed to the heterogeneity.

### Sensitivity analysis

**Figure 4:**
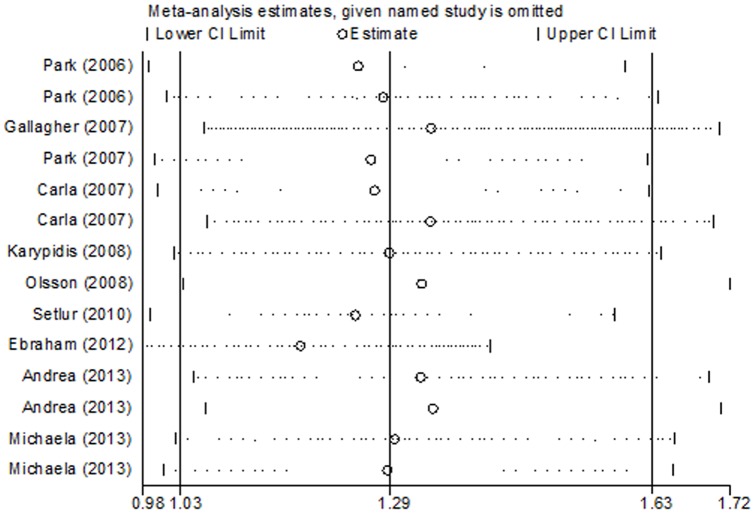
Influence analysis for the recessive model in the overall meta-analysis. The figure shows the influence of individual studies on the summary OR.

Sensitivity analysis was performed to distinguish each study's influence on the pooled OR by repeating the meta-analysis while omitting each study one at a time [28]. The results demonstrated that no individual study significantly affected the pooled OR, indicating our results are reliable and robust (Figure 4). Furthermore, when the studies that did not have data for all three genotypes were excluded from the analysis, the estimated pool OR still did not change significantly.

### Publication bias

**Figure 5:**
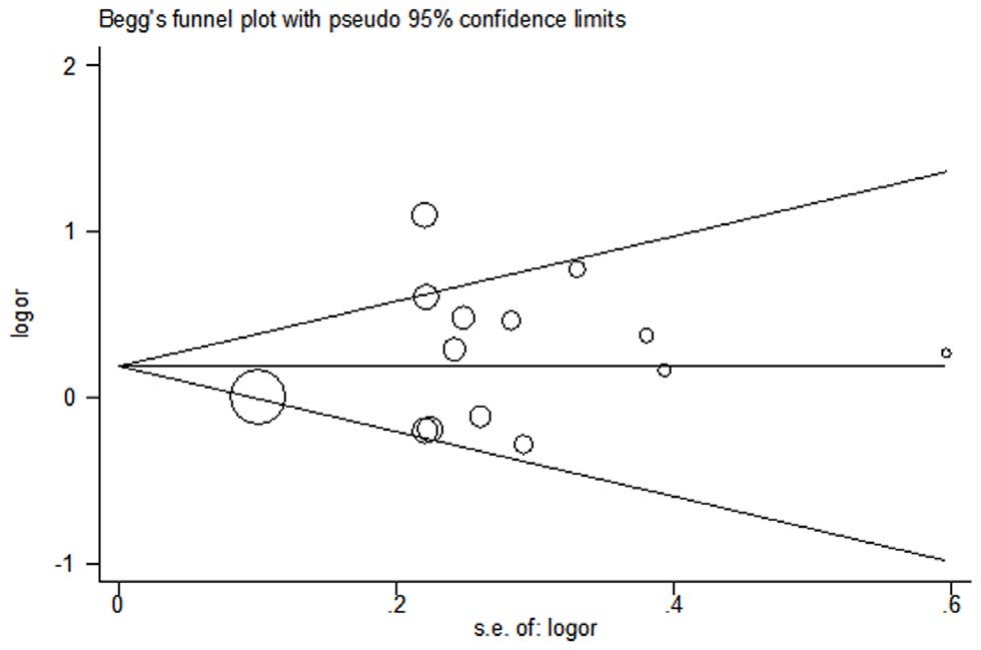
Begg's funnel plot of publication bias test. Each point represents an isolated study for the indicated association. Log (OR): natural logarithm of OR. *Horizontal line* mean effect size.

Begg's funnel plot and Egger's test were performed to assess the publication bias and the results are shown in Figure 5. The results of the funnel plots did not show any evidence of obvious asymmetry (*t* = 1.07, *P* = 0.31 for recessive model).

## Discussion

It has been suggested that glucuronidation is a major detoxification pathway for compounds like bilirubin and steroid hormones, as well as xenobiotics, including alcohol, and environmental carcinogens. During tumorigenesis progression, UGT2B17 acts as a catalyzer to transfer glucuronic acid from UDPGA into endogenous and exogenous molecules. Altered expression of the UGT2B17 protein is mediated by its gene deletion polymorphism, and has been shown to be relevant to carcinogenesis. Correlations between the *UGT2B17* deletion polymorphism and tumors have attracted the attention in the research community. Park *et al*. [17] found that the *UGT2B17* null polymorphisms may modify the risk of prostate tumor, particularly among those who have a family history of the disease. Interestingly, Carla *et al.* found significant differences in women but not in men, especially susceptibility for lung adenocarcinoma, which may be explained by its decreased rates of 4-(methylnitrosamino)-1-(3-pyridyl)-1-butano (NNAL) glucuronidation in women. However, a study conducted by Gallagher *et al.* demonstrated that there was no association between the *UGT2B17* deletion polymorphism and risk of prostate tumor in Caucasian men.

In general, the results depicting the association between the *UGT2B17* deletion polymorphism and tumor risk remains conflicting and inconclusive. These conflicting findings may be a result of the small effect of the *UGT2B17* deletion polymorphism on tumor risk, or the low statistical power in the published studies. Therefore, the meta-analysis presented herein, including 5,733 cases and 5,112 controls from 14 case-control studies, provides little evidence of an association between the UG2B17 deletion polymorphism and tumor risk. The findings of the meta-analysis are unclear; when all the studies in the analysis, a small but significant decrease of tumor risk were reported in the recessive model, but with great heterogeneity. The other three models did not show positive effects of tumor risk reduction. When stratified by the source of control material, meaningful results were only observed in the hospital-based controls with OR = 1.46, 95% = 1.06–2.01 and *P* = 0.11. Recent studies indicate that men exhibited a 4-fold higher UGT2B17 expression level than women, and these findings are indicate that this increase gene expression may play a greater role in reducing tumor risks in men than in women. By contrast, our analysis found out that this polymorphism did not have any gender-specific effects on tumor risks (*P* = 1.19, 95%CI = 0.95–1.48 for men and *P* = 1.53, 95%CI = 0.85–2.75 for women).

Although, the results did report some possible links in tumors, the positive results were only slightly more significant than what would be found by chance. Several studies had merit and deserve individual mention. Owing to the limitations of the genotyping methods utilized in the early published studies, the heterozygous (Del/Ins) and homozygous (Ins/Ins) UGT2B17 genotypes could not be distinguished [15], [17], [19], [22]. Nonetheless, these studies claim that a difference was observed when comparing individuals with at least one *UGT2B17* allele to those with *UGT2B17* homozygous deletions [13], [29]. As a result, the precise function of the *UGT2B17* deletion polymorphism has tumor risk could not be revealed using the recessive model.

*I^2^* values were used to demonstrate heterogeneity and great heterogeneity was observed with the recessive model (*I^2^* = 67.3%), which indicates that our meta-analysis may be inaccurate. The heterogeneity may emanate from various factors, such as population characteristic diversities, DNA extraction materials used and differences in controls. Tumor cells typically lose heterozygosity [30], and it has been hypothesized that they regularly affect the proline allele in squamous cell carcinomas [31]. Likewise, loss of heterozygosity has also been found in exfoliated cells [32]. Accordingly, DNA extracted from whole blood cells should be used for detecting genetic polymorphisms rather than tumor issue. One study that extracted DNA both from blood and paraffin-embedded tissue specimens showed an association between the *UGT2B17* null genotype and breast tumors [22]. Moreover, we conducted a meta-regression (*P_material_ = *0.02) and Galbraith radial plots (Figure 3A) to discover the source of heterogeneity. The results show that this study is a key spring of the analysis's heterogeneity. Thus, we excluded this research and the *I^2^* value was measured again. The results remained the same with *I^2^* = 44% and the *P_source of control_* = 0.084 when meta-regression was conducted, which meant heterogeneity still existed.

To sort out the possible causes of heterogeneity, the control selection procedures in the pooled studies were taken into consideration. The results show that the source of control will affect the meta-analysis [33]. Different control sources may impact the conclusion of our study through the case-control comparison procedure [34]. Some of the pooled studies utilized a healthy population as the reference group, while others used hospitalized patients. Hospital-based controls may not correlate well with the general population, especially when the explored genotypes were involved in the disease conditions [35]. Therefore, we performed a subgroup analysis by control sources to eliminate the interference from this factor. When all the studies were used in the analysis, we found positive results in the hospital-based controls but not among the population-based controls (*P* = 1.46, 95%CI = 0.85–2.75). Following exclusion of the breast tumor study [22] and one heterogeneous study [15], a large heterogeneity was noted among the hospital-based controls (Figure 3B). A possible explanation for this result is that the *UGT2B17* polymorphism may influence the susceptibility to non-tumor diseases, so its genotype frequency possibly differed between the hospital-based and population-based controls. Proper population-based control subjects may reduce the biases in the genetic association studies rather than the hospital controls which could be relatively easier and more economical to recruit. The results presented herein are more reliable when two studies were excluded for homogeneity [15], [22]. In conclusion, our meta-analysis did not demonstrate a significant role of the *UGT2B17* deletion polymorphism and tumor susceptibility (OR = 1.118, 95%CI = 0.938–1.332, *P*>0.1).

To the best of our knowledge this is the first meta-analysis that did a comprehensive assessment of the relationship between the *UGT2B17* deletion polymorphism and the risk of tumor. Neither a total analysis, nor a stratified investigation, demonstrated a significant association with susceptibility to tumor; which means the *UGT2B17* deletion polymorphism may have no role in tumor vulnerability. However, because of the relatively small sample sizes and the inadequate numbers of studies, the results need to be further validated and confirmed. Our analysis had other limitations.: First, our results were based on an unadjusted estimated, a more precise analysis would have been conducted if more detailed individual data were available which could have been adjusted for age or gender Second, only studies with full text from English databases were selected, and this may have led to publication. Third, the majority studies used were investigations in Europeans. However, our meta-analysis also had some merits. When two studies were excluded from the analysis, the homogeneity of pooled studies was maintained, which guaranteed reliability of our analysis. Additionally, no differences in tumor susceptibility between men and women have been discovered and it might be worth to note that sex hormones may contribute to the tumor in other ways.

## Conclusion

This study reveals no significant association between the *UGT2B17* deletion polymorphism and tumor risk. Moreover, it would be interesting to extend the investigation to a wider range of human populations which may result in a better, comprehensive understanding of the association.

## Supporting Information

Checklist S1 **PRISMA 2009 checklist.**
(DOC)
